# Draft Genome Sequence of Stutzerimonas stutzeri NT-I, Which Reduces Selenium Oxyanions into Elemental Selenium and Volatile Selenium Species

**DOI:** 10.1128/mra.01016-22

**Published:** 2022-11-03

**Authors:** Masashi Kuroda, Kazunari Sei, Mitsuo Yamashita, Michihiko Ike

**Affiliations:** a Department of Social and Environmental Studies, Faculty of Social and Environmental Studies, Tokoha University, Shizuoka, Japan; b Department of Health Science, School of Allied Health Sciences, Kitasato University, Kanagawa, Japan; c Department of Applied Chemistry, Faculty of Engineering, Shibaura Institute of Technology, Tokyo, Japan; d Division of Sustainable Energy and Environmental Engineering, Graduate School of Engineering, Osaka University, Osaka, Japan; University of Southern California

## Abstract

Stutzerimonas stutzeri strain NT-I effectively reduces selenate and selenite into elemental selenium and volatile selenium species. It is thus a promising biological agent for treatment of selenium-contaminated wastewater. We here report the draft genome sequence of this strain.

## ANNOUNCEMENT

Selenium (Se) is an industrially important element as a key component of semiconductor materials. It is also a known environmental water contaminant because Se oxyanions, selenate (SeO_4_^2−^) and selenite (SeO_3_^2−^) are toxic and highly soluble in water. The utilization of Se-metabolizing bacteria that can reduce selenate and selenite into insoluble or volatile Se species is a promising option for the treatment of Se-contaminated wastewater (1).

Stutzerimonas stutzeri NT-I was originally isolated from drainage water from a Se refinery plant (2). Under aerobic conditions, strain NT-I reduced highly concentrated selenate and selenite into elemental Se nanoparticles, which are insoluble in water (2). Strain NT-I also synthesized volatile methylated Se species at the highest rate among Se-volatilizing microorganisms reported to date (3). Taking these characteristics into account, *S. stutzeri* NT-I is a promising candidate biological agent for treatment of wastewater containing high concentrations of Se. Therefore, the molecular mechanisms of selenium metabolism by strain NT-I are of great interest.

The genomic DNA of strain NT-I was extracted with the following procedure based on Marmur’s method (4) from cells aerobically cultured in 50 mL of BD Bacto tryptic soy broth (Thermo Fisher Scientific, MA, USA) at 28°C for 24 h. The cells were collected by centrifugation (10,000 × *g*, 4°C, 10 min) and resuspended in 10 mL of cell suspension buffer (10 mM Tris-HCl [pH 8.0], 1 mM EDTA-Na_2_, 350 mM sucrose). The cell suspension was mixed with 5 mL of lysing solution (100 mM Tris-HCl [pH 8.0], 300 mM NaCl, 20 mM EDTA-Na_2_, 2% sodium dodecyl sulfate, 100 μg/mL proteinase K) and incubated at 55°C for 1 h. The lysate was purified with phenol-chloroform extraction twice, and the supernatant was transferred to a new plastic tube. Isopropanol of a 60% volume of the recovered supernatant was gently added into the tube. DNA was rolled up with a glass rod from the boundary of the water and isopropanol phases and resuspended in TE buffer (10 mM Tris-HCl, 1 mM EDTA-Na_2_) containing 100 μg/mL RNase A. After incubation at 37°C for 10 min, the DNA was purified with phenol-chloroform extraction, chloroform–3-methyl-1-butanol extraction, and ethanol precipitation. Obtained DNA was suspended in ultrapure water and subjected to genome sequencing. A shotgun genomic library was prepared using a Roche/454 GS Rapid library prep kit (Hoffmann-La Roche, Basel, Switzerland). Sequencing was performed using a Roche 454 FLX genome sequencer (Roche). A total of 63,558,482 bp of reads with an average read length of 389.30 bp were obtained, corresponding to 13× genome coverage. Quality control and assembly of raw reads were conducted using Newbler version 2.3 (Roche). The draft genome sequence consisted of 115 contigs, a GC content of 64.0%, totaling 4,833,352 bp, an *N*_50_ statistic of 135,402 bp, and a maximum contig size of 218,194 bp. A total of 4,520 coding sequences (CDSs) and 54 RNAs were predicted by the RAST Prokaryotic Genome Annotation Server (5). Default parameters were used for all software.

### Data availability.

The draft genome sequence of *S. stutzeri* NT-I has been deposited in NCBI/ENA/DDBJ under accession numbers BCAJ01000001 to BCAJ01000115. Raw reads are available under accession number DRA014746. BioProject and BioSample accession numbers are PRJDB4190 and SAMD00040029, respectively.
